# Erysipelas of the right arm due to *Bordetella trematum*: a case report

**DOI:** 10.1186/s13256-021-02896-1

**Published:** 2021-07-13

**Authors:** M. Lacasse, K. Inyambo, A. Lemaignen, M. Mennecart, S. Gensburger, A. S. Valentin, L. Bernard, B. Fougère

**Affiliations:** 1 Geriatric Department, Tours University Hospital, Tours, France; 2 Bacteriology Department of Tours University Hospital, Tours, France; 3 Infectious Diseases Unit, Tours University Hospital, Tours, France

**Keywords:** *Bordetella trematum*, Opportunistic infection, Skin and soft tissue infection, Case report

## Abstract

**Background:**

*Bordetella trematum* is unknown to most clinicians and microbiologists. However, this Gram-negative opportunistic bacterium can be responsible for ulcer superinfection but also bacteremia and sometimes death by septic shock.

**Case report:**

We report the case of erysipelas due to *B. trematum* with bacteremia in an immunocompromised 88-year-old Caucasian patient.

**Conclusion:**

In immunocompromised patients, unusual microbial agents such as *B. trematum* can be responsible for cutaneous and systemic infections, requiring specific antibiotic therapy. Therefore, clinicians should be aware of the need for specific bacterial identification such as matrix-assisted laser desorption ionization time-of-flight mass spectrometry and 16S ribosomal RNA sequencing in the context of atypical evolution of erysipelas in such patients.

**Supplementary Information:**

The online version contains supplementary material available at https://doi.org/10.1186/s13256-021-02896-1.

## Introduction

The genus *Bordetella* belongs to the *Alcaligenaceae* family and includes dozens of species [1, 2] mostly responsible for bronchopulmonary infections in mammals. The agent of whooping cough, *Bordetella pertussis,* is the most famous of *Bordetella* species. *Bordetella trematum* was identified in 1996 from chronic media otitis and chronic ulcer [3]. This germ is difficult to isolate, and its pathogenicity remains debated in humans. An increasing number of infections due to *B. trematum* are described in the literature as a potential emerging pathogen. In this context, we report the case of erysipelas due to *B. trematum* associated with bacteremia.

## Case

**Fig. 1:**
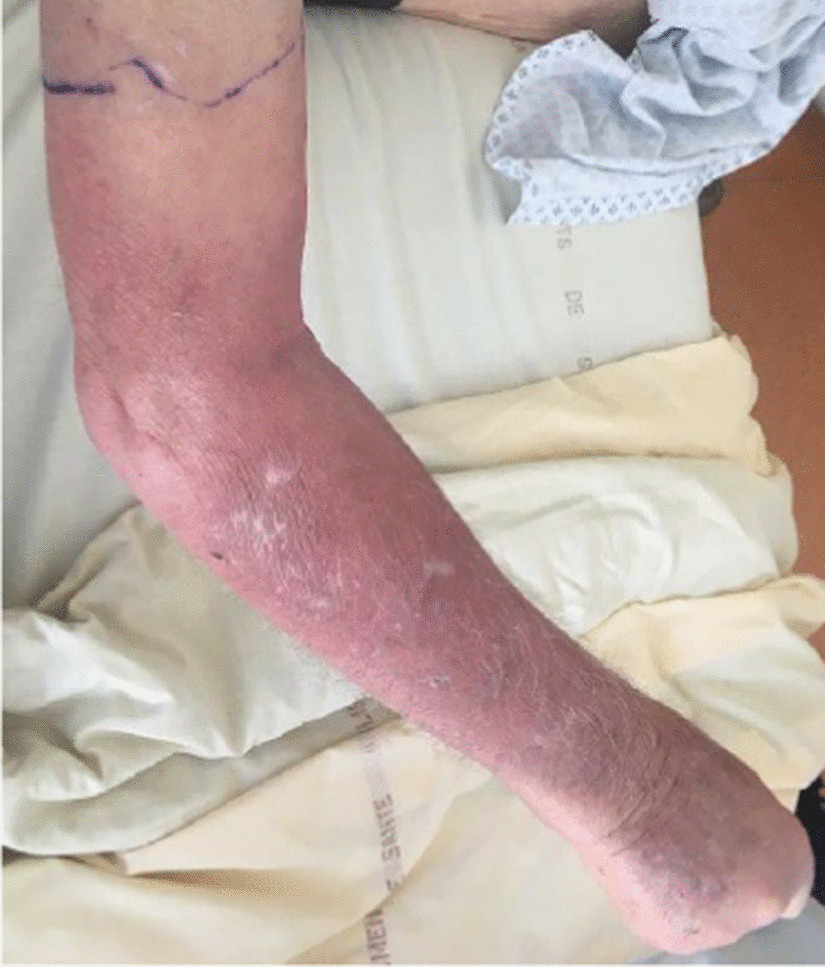
Cellulitis due to *Bordetella trematum*

**Fig 2:**
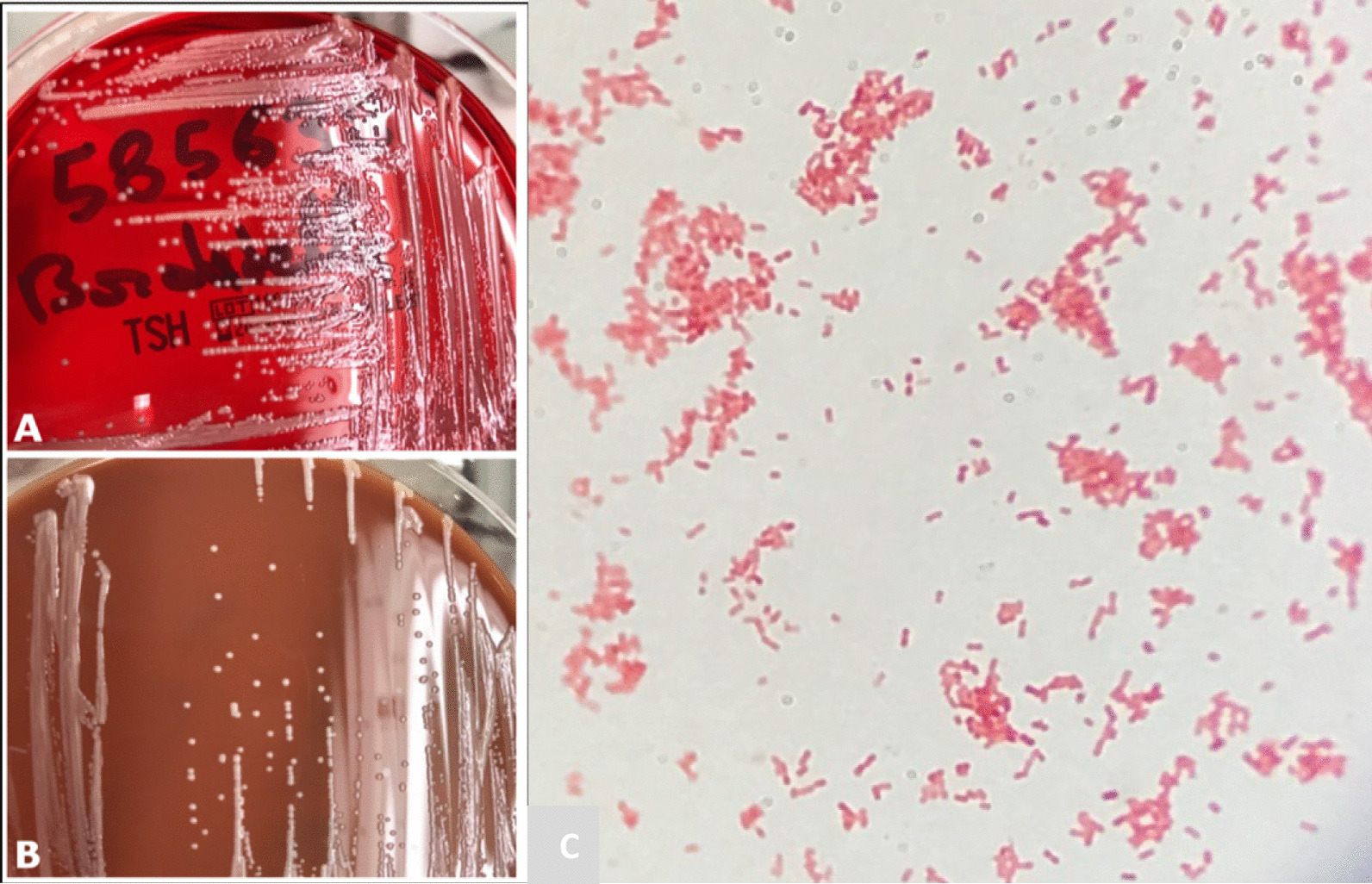
Growth of *Bordetella trematum* in blood agar plate (**a**) and chocolate agar (**b**). Gram coloration of *Bordetella* (**c**)

An 88-year-old Caucasian male patient had a history of chronic kidney failure, stress angina with quadruple coronary bypass surgeries, removal of multiple skin carcinomas, and chronic lymphocytic leukemia treated for a year by chloraminophene in 2012. This former carpenter, who normally lived alone, presented himself to emergency unit for repeated falls and erythematous edema of the right arm since 3 days suggesting erysipelas (Fig. 1). On admission, his temperature was 39.7 °C, his pulse was at 107 beats per minute, and blood pressure was 131/54 mmHg. He presented erysipelas of the right arm with edema and without any adenopathy. There was no other clinical sign at admission. The blood test found leukocytes at 156 × 10^9^/L consisting of 12.8 × 10^9^/L neutrophils and 140 × 10^9^/L lymphocytes, serum biochemical analysis with creatinine level at 160 µmol/L, hyperkalemia at 5.3 mmol/L, and elevated C-reactive protein (CRP) at 271 mg/L. One set of blood culture was collected from a peripheral access prior to administration of amoxicillin, and the patient was transferred to the geriatric unit. Aerobic blood culture grew with small, gray, shiny, rounded colonies in polyvitex and blood agar plate (Fig. 2a, b), which were Gram-negative coccobacilli (Fig. 2c) after 18 h at 35° CO_2_ on conventional medium. *B. trematum* was identified using matrix-assisted laser desorption ionization time-of-flight mass spectrometry (MALDI-TOF MS) and confirmed by 16S ribosomal RNA sequencing (Additional file 1). Another blood culture was performed 2 days after the first one and while taking antibiotic therapy and was negative. No additional tests were carried out. Diagnosis of thrombosis was eliminated by venous ultrasonography and Doppler of his right arm.

After a multidisciplinary meeting involving geriatricians, infectious diseases specialists, and microbiologists, antibiotic therapy was changed to ceftazidime 1 g per day for 10 days after a charging dose of 2 g, slow intravenous, according to susceptibility testing (Table 2) and renal function at that time. The clinicobiological evolution was rapidly favorable. The patient was discharged from hospital and referred to hematologists on the basis of white blood cell count, and finally, the chemotherapy by chloraminophene was resumed. He was rehospitalized for depression the next month, at which point his arm was totally healed. At the last follow-up, 6 months after the first symptoms, the patient was in good health and has not had any recurrence of infection.

## Discussion

Our case illustrates an atypical case of erysipelas in an immunocompromised patient. Identification of *B. trematum* required MALDI-TOF and 16S-rtPCR sequencing, and lead to modification of empiric antibiotic treatment for ceftazidime with good evolution.

*Bordetella trematum* is a small, mobile, capsulated, nonsporulating, and nonfermentative Gram-negative bacillus. The term *trema* refers to something pierced or penetrated like wounds, and inspired its name [1]. Little is known about its pathophysiology because it is scarce and quite recently identified. It is genetically close to *B. avium* and frequently mistaken for it [4]. The supposed ecological reservoir of *B. trematum* is soil [5], and most cases involve skin and soft-tissue infections. Chang *et al.* have revealed a cytolethal distending toxin (CDT) [6]. This genotoxin has only been described in *B. trematum*, which may partly explain the ineffectiveness of antibiotic treatment in some patients. It could be interesting to better characterize the role of this genotoxin in the specific virulence of *B. trematum*, and evaluate the specific effect of antitoxin antibiotic drugs such as macrolides.

The interpretation of a positive blood culture of *B. trematum* remains difficult and, in the context of sepsis, should prompt a suitable antibiotic therapy. Thanks to new diagnostic tools such as MALDI-TOF and molecular biology, it is now easier to identify [7–12], though there are still mistakes in its recognition.

*Bordetella trematum* infections have been identified in 14 case reports in the literature (12 are summarized in Table 1). Most cases were found in diabetic patients, particularly from ulcers and chronic ear infections [1, 4, 7, 9, 12]. Several authors consider that this germ is not very pathogenic and that its natural evolution does not require specific antibiotherapy [4, 7].

**Table 1:** Case reports of *Bordetella trematum* infections in human

Author	Sex/age (year)	Diagnosis	Immunocompromised	Comorbidity	Microbiological isolation	Growth delay (hour)	Associated germ	Identification method	Treatment	Duration of treatment (days)	Outcome
Y Castro *et al.* 2019	F/74	Infected ulcer and necrosis skin	Renal failure	Diabetes mellitus	Local sample	24	*Enterococcus faecalis*, *Stenotrophomonas maltophilia*	VITEK + PCR 16S	Surgical debridement tazocillin next Meropenem/levofloxacin	19	Death
Desurmont *et al.* 2018	F/65	Bacteremia on chest bleeding from metastasis	Metastatic breast cancer	0	Blood culture	24	*E. faecalis*	MALDI TOF	Tazocillin	21	Healing
Majewski *et al.* 2016	M/61	Septic shock from soft-tissue infection	Renal failure	Diabetes mellitus, coronary failure	Blood culture	24	0	PCR 16S	Ciprofloxacin/clindamycin/tobramycin	7	Death
Almagro-Molto *et al.* 2015	M/65	Infected ulcer	0	Diabetes mellitus, vascular failure	Local sample	48	*Morganella morganii*, *E. faecalis*, *Staphylococcus aureus*, *Proteus vulgaris*	MALDI TOF + PCR 16S	Ciprofloxacin	3	Persistent infection
Almagro-Molto *et al.* 2015	F/72	Osteomyelitis	0	Diabetes mellitus	Local sample	48	*S. maltophila, E. faecalis,* SARM	MALDI TOF + PCR 16S	Tazocillin next meropenem	14	Favorable evolution
Almuzara *et al.* 2015	M/14	Chronic hip osteomyelitis	0	0	Bone biopsy	NA	*Escherichia coli*	VITEK + MALDI TOF + PCR 16S	Meropenem/Bactrim/surgical debridement	180	Healing
Saksena *et al.* 2015	F/0,6	Bacteremia and delayed development	0	0	Blood culture	72	0	VITEK + PCR 16S	Ciprofloxacin/azithromycin	> 5	Healing
Halim *et al.* 2014	M/60	Septic shock from bacteremia	Burned (60% of body)	0	Blood culture	48	*Enterobacter cloacae*	PCR 16S	Imipenem/netilmicin/colistin	1	Death
Tena *et al.* 2015	F/54	Secondary peritonitis	0	0	Peritoneal fluid	48	*Klebsiella pneumoniae*, *Xanthobacter* species	MALDI TOF + PCR 16S	Tazocillin	5	Healing
Hernandez-Porto *et al.* 2013	F/76	Infected ulcer	Renal failure	Diabetes mellitus, vascular failure	Local sample	NA	*Achromobacter xylosoxidans*	VITEK	Bactrim/ceftazidime	21	Favorable evolution
Daxboeck *et al.* 2004	M/82	Infected ulcer	0	Diabetes mellitus	Chirurgical sample	NA	0	PCR 16S	Surgical debridement	0	Favorable evolution
Vandamme *et al.* 1996	M/29	Chronic otitis medium	NA	NA	Local sample	NA	*Pseudomonas aeruginosa*, SCN	PCR 16S	Clindamycin	7	Healing

A. xyloxydans: Achomobacter xyloxydans; E. faecalis: Enterococcus faecalis; E. cloacae: Enterobacter cloacae; E. coli: Escherichia coli ; K. pneumoniae: Klebsiella pneumoniae; M. morganii: Morganella morganii; P. vulgaris: proteus vulgaris; P. aeruginosa: Pseudomonas aeruginosa; SCN: Staphylococcus coagulase negative; S. aureus: Staphylococcus aureus; SARM: Staphylococcus resistant meticillin; S. maltophilia: Stenotrophomonas maltophilia; F : female; M: male; MALDI TOF: Matrix-Assisted Laser Desorption Ionization Time-Of-Flight; PCR 16S: 16S Ribosomal RNA Sequencing

In contrast, as reported with our patient, invasive infection seems to occur mostly in immunocompromised patient [8, 13, 14]. Thereby, *B. trematum* could be considered as an opportunistic agent. However, some cases are reported in immunocompetent patients such as in a 7-month-old child [11] or in a 54-year-old patient with peritonitis [10].

Once the bacteria have been identified, treatment is not consensual. Indeed, there are no data about the antibiotic susceptibility of *B. trematum* in European guidelines (European Committee on Antimicrobial Susceptibility Testing, EUCAST 2018). We have reported in Table 2 the different antibiotic susceptibility tests described in the literature. There were interpreted according to minimal inhibitory concentration (MIC) interpretative standards of closely related species (other non-*Enterobacteriaceae* and *Enterobacteriaceae*). The choice of ceftazidime in our case was made according to the sensitivity testing. Despite the piperacillin–tazobactam sensitivity *in vitro* in all precedent case reports, patients died despite piperacillin–tazobactam therapy in two of them [12, 13] and had an unfavorable outcome in another one [11], whereas, in the case of Hernandez-Porto *et al.*, the patient had also renal failure and the treatment was a success. Given these discordant results, the place of piperacillin–tazobactam in the therapeutic strategy for the treatment of *B. trematum* infections should be reconsidered.

**Table 2:** Antibiotic susceptibility testing of *Bordetella trematum* in the literature

Antibiotics	Actual reported case	Y Castro *et al.* 2019	Desurmont *et al*. 2018	Saksena *et al.* 2015	Almagro-Molto *et al.* 2015 case 1	Almagro-Molto *et al*. 2015 case 2	Almuzara *et al.* 2015	Halim *et al.* 2014	Majewski *et al.* 2016	Tena *et al*. 2015	Hernandez-Porto *et al.* 2013
Ampicillin	–	–	S	–	S	S	I	–	–	R	–
Ampicillin + clavulanic acid	–	–	S	S	S	S	I	R	S	S	S
Piperacillin	S	–	–	I	S	S	–	–	–	–	–
Piperacillin + tazobactam	S	S	S	S	S	S	S	–	S	S	S
Cefoxitin	–	–	R	–	R	R	–	–	–	–	–
Cefotaxime	R	R	–	–	R	R	R	R	I	R	R
Cefuroxime	–	–	R	–	R	R	–	–	R	–	R
Ceftazidime	S	R	–	R	R	R	S	R	S	–	S
Cefepime	S	S	–	I	S	S	S	–	–	R	–
Ceftriaxone	–	R	R	I	–	–	–	–	–	–	–
Aztreonam	R	R	R	–	–	–	–	–	–	–	R
Imipenem	S	S	–	S	S	S	S	S	S	S	S
Meropenem	S	S	–	R	S	S	S	–	–	S	S
Levofloxacin	–	S	–	I	S	S	–	–	S	S	–
Ciprofloxacin	–	I	–	S	R	R	I	S	S	S	R
Gentamicin	I	S	–	R	–	–	S	R	R	S	S
Tobramycin	S	S	–	I	S	S	–	R	S	S	–
Netilmicin	R	–	–	–	–	–	–	S	–	–	–
Amikacin	S	S	–	R	S	S	S	R	S	S	S
Tigecycline	–	S	–	–	S	S	–	–	–	–	–
Minocycline	S	–	–	–	S	S	–	–	–	–	–
Tetracycline	–	–	S	–	–	–	–	S	–	–	–
Colimycin	S	–	–	R	–	–	S	S	–	–	–
Fosfomycin	–	–	R	–	R	R	–	–	–	–	–

S: susceptible; I: intermediate; R: resistant

Furthermore, clinicians should remain cautious, especially in immunocompromised patient, when presented with atypical skin infection.

## Conclusion

*Bordetella trematum* can be considered as an opportunistic agent scarcely described but with a pathogenicity not to be neglected because of its wide range of severity: from simple colonization to septic shock. This agent, which is increasingly identified, could be considered as a potential emerging pathogen. Therefore, clinicians should be aware of the need for specific means of bacterial identification such as MALDI-TOF and 16S-rtPCR in the context of complicated evolution of erysipelas or associated unidentified Gram-negative bacteremia in immunocompromised patients.

## Supplementary Information

**Additional file 1.** Annex: result of 16S ribosomal RNA sequencing.

## Data Availability

Data sharing is not applicable to this article, since no datasets were generated or analyzed during the current study.

## Abbreviations

*B. trematum*: *Bordetella trematum*
16S-rtPCR: 16S ribosomal RNA sequencing
MALDI-TOF MS: Matrix-assisted laser desorption ionization time-of-flight mass spectrometry
CDT: Cytolethal distending toxin
EUCAST: European Committee on Antimicrobial Susceptibility Testing
Piperacillin–tazobactam: Tazocillin
MIC: Minimal inhibitory concentration

## References

[1] Vandamme P, Heyndrickx M, Vancanneyt M, Hoste B, De Vos P, Falsen E,* et al*. *Bordetella trematum* sp. nov., isolated from wounds and ear infections in humans, and reassessment of *Alcaligenes denitrificans* Rüger and Tan 1983. Int J Syst Bacteriol. 1996;46(4):849–58. 10.1099/00207713-46-4-849 8863408

[2] Novikov A, Marr N, Caroff M. A comparative study of the complete lipopolysaccharide structures and biosynthesis loci of *Bordetella avium*, *B. hinzii*, and *B. trematum*. Biochimie. 2018;159:81–92. 10.1016/j.biochi.2018.12.011 30578925

[3] Dorittke C, Vandamme P, Hinz KH, Schemken-Birk EM, Wirsing von König CH. Isolation of a *Bordetella avium*-like organism from a human specimen. Eur J Clin Microbiol Infect Dis. 1995;14(5):451–4. 10.1007/BF02114904 7556237

[4] Hernández-Porto M, Cuervo M, Miguel-Gómez MA, Delgado T, Lecuona M. Diabetic leg ulcer colonized by *Bordetella trematum*. Rev Esp Quimioter. 2013;26(1):72–3. 23546468

[5] Hamidou Soumana I, Linz B, Harvill ET. Environmental origin of the genus *Bordetella*. Front Microbiol. 2017. 10.3389/fmicb.2017.00028. PMC5258731 28174558

[6] Chang D-H, Jin T-E, Rhee M-S, Jeong H, Kim S, Kim B-C. Draft genome sequence of *Bordetella trematum* strain HR18. Genome Announc. 2015. 10.1128/genomeA.01357-14. PMC4290983 25573930

[7] Daxboeck F, Goerzer E, Apfalter P, Nehr M, Krause R. Isolation of *Bordetella trematum* from a diabetic leg ulcer. Diabet Med. 2004;21(11):1247–8. 10.1111/j.1464-5491.2004.01310.x 15498093

[8] Halim I, Ihbibane F, Belabbes H, Zerouali K, Mdaghri NE. Isolation of *Bordetella trematum* from bacteremia. Ann Biol Clin. 2014;72(5):612–4. 10.1684/abc.2014.0998 25336136

[9] Almagro-Molto M, Eder W, Schubert S. *Bordetella**trematum* in chronic ulcers: report on two cases and review of the literature. Infection. 2015;43(4):489–94. 10.1007/s15010-014-0717-y 25600927

[10] Tena D, Medina MJ, Sáez-Nieto JA. Isolation of *Xanthobacter* species and *Bordetella trematum* in a patient with polymicrobial peritonitis. Infect Dis Clin Pract. 2017;25(5):e16–9.

[11] Saksena R, Manchanda V, Mittal M. *Bordetella trematum* bacteremia in an infant: a cause to look for. Indian J Med Microbiol. 2015;33(2):305–7. 10.4103/0255-0857.154891 25865992

[12] y Castro TR, Martins RCR, Dal Forno NLF, Santana L, Rossi F, Schwarzbold AV,* et al*. *Bordetella trematum* infection: case report and review of previous cases. BMC Infect Dis. 2019;19(1):485. 10.1186/s12879-019-4046-8 PMC6543606 31146691

[13] Majewski LL, Nogi M, Bankowski MJ, Chung HH. *Bordetella trematum* sepsis with shock in a diabetic patient with rapidly developing soft tissue infection. Diagn Microbiol Infect Dis. 2016;86(1):112–4. 10.1016/j.diagmicrobio.2016.05.019 27397578

[14] Desurmont-Dupas MC, Cattoën C, Bonnet I. Bactériémie à *Bordetella trematum* dans un contexte d’immunosuppression [Bordetella trematum bacteremia in an immunosuppressed patient]. Med Mal Infect. 2018;48(8):547-9. 10.1016/j.medmal.2018.03.008. 30190162

