# Supplementary figures and images for: Erysipelas of the right arm due to Bordetella trematum: a case report
Source: J Med Case Rep. 2021 Jul 13;15:365. doi: 10.1186/s13256-021-02896-1 (PMC8276433; doi:10.1186/s13256-021-02896-1)

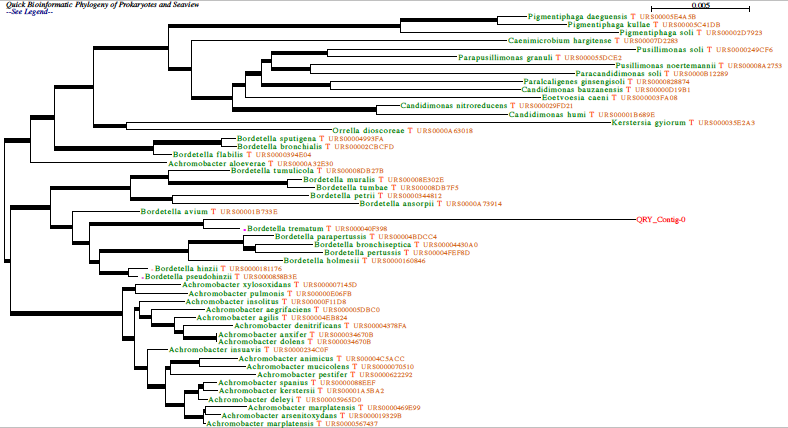


Annex : Result of 16S ribosomal RNA sequencing.

Supplement: Supplementary file 1 — Additional file 1. Annex: result of 16S ribosomal RNA sequencing. [file 13256_2021_2896_MOESM1_ESM.docx]
